# Attracting adolescents to become doctors and nurses: differential importance of personal and environmental factors in 61 economies

**DOI:** 10.1186/s12960-023-00823-7

**Published:** 2023-05-15

**Authors:** Luyang Guo, Kit-Tai Hau

**Affiliations:** Department of Educational Psychology, The Chinese University of Hong Kong, Hong Kong SAR, P.R. China

**Keywords:** Health career, Adolescent, Government expenditure, Working conditions

## Abstract

**Background:**

Doctors and nurses play a fundamental role in maintaining global health systems and achieving universal health care coverage. However, significant shortages persist, and little is known about the popularity of these careers among young people in various economies or the relative impact of personal inputs and contextual factors.

**Methods:**

Using data from the large-scale Programme of International Student Assessment (PISA) 2018, we showed the recent distribution of adolescents' medical (doctor) and nursing career expectations in 61 economies. With multilevel logistic and hierarchical linear regression, we examined the relative importance of economic indicators, health work conditions, and personal background factors in affecting adolescents' health career expectations.

**Results:**

Approximately 11% of adolescents expected to be doctors in each economy, while only 2% expected to be nurses. Adolescents were attracted to health professions mainly by system-level favourable conditions (accounting for 1/3 variance), including (a) government health expenditure beyond that expected gross domestic product (GDP); (b) a safe working environment for doctors in wealthy nations; and (c) high salaries for nurses in less-developed economies. In contrast, adolescents' background (gender, social status, and academic ability) was less influential, explaining only 10% of the differences.

**Conclusions:**

In the technological and digital era, high-ability students are equally competitive for emerging careers other than doctors and nurses. In developing countries, a high salary package and societal respect are enough to attract adolescents to nursing careers. In contrast, for developed countries, extra expenditures beyond regular GDP allocation and a safe work environment are crucial in attracting adolescents to become doctors. Salary may effectively attract international-trained doctors and nurses, but the work environment will likely emerge as an essential factor in retaining migrants in their positions.

*Trial registration number*: No human participants were involved in this study.

**Supplementary Information:**

The online version contains supplementary material available at 10.1186/s12960-023-00823-7.

## Introduction

An adequate, motivated, and evenly distributed health workforce is essential to attain universal health coverage and realize the United Nations Sustainable Development Goals. However, the Global Strategy on Human Resources for Health: Workforce 2030 estimated a global shortage of 18 million health workers by 2030 [1]. With more recent data, the projected shortage has been narrowed to 10 million, but profound inequalities were highlighted as being exacerbated in less-developed African and Eastern Mediterranean regions [2]. To ensure sustainable health workforce planning, recruitment, and retention are three essential elements [3]. Enhancing the "pull" factors that attract young people to health professions is indispensable at the planning stage. Little is known about whether some factors are more generally applicable to young people in all economies, while some are only specific to developing or developed economies. The present research used a large-scale international study to examine the impacts of various push and pull factors at the system and personal levels in 61 economies.

Despite substantial growth in health workers during the COVID-19 pandemic, shortages persist in many countries for two reasons. On the supply side, an insufficient number of students expect health careers, and many medical school graduates are undecided about pursuing a career in health. In Vietnam, only one-fourth of medical school graduates intend to stay in the field [4]. Approximately 13.7% of Chinese undergraduate medical students intend to withdraw from the profession [5]. A review of 625 studies reported an average attrition rate of 9.1% among medical school graduates worldwide [6]. Many developed economies, such as Canada, the UK, and the USA, do not have a sufficient internal supply of such medical professionals and thus heavily rely on internationally trained doctors and nurses to staff their health systems. This outflow from disadvantaged economies worsens their originally underprovided expertise and resource problems [7]. If we were to reduce the harm to developing economies, more advanced economies would in turn reduce the in-migration of trained health workers from less-developed economies. Therefore, developed economies must spend more effort increasing their domestic training and retaining their health workforce.

On the demand side, some governments suffer from financial difficulties in paying the attractive remuneration package necessary to recruit enough health workers. Other governments may lack full-time vacancies for which to recruit new graduates, reducing the attractiveness of these training programmes [8]. Many planning, recruitment, and retention suggestions have been provided to solve shortages and inequalities, especially for remote rural areas [9, 10]. Nonetheless, relatively less attention has been given to cultivating students' health career interests at an earlier stage.

Adolescents' career expectations, which indicate their realistic and accessible occupational choices, resemble the labour market job distribution [11]. Adolescence—age 10 to 19—is a critical stage when individuals begin to understand the complex nature of occupational choices and social barriers to pursuing their ideal careers [12, 13]. Previous studies have found that young people make necessary career adaptations due to perceived social norm constraints and insufficient environmental support [14]. For instance, boys may avoid nursing careers owing to gender stereotypes, and low socioeconomic status (SES) students may be deterred from pursuing medical careers because of high tuition fees [15, 16]. Importantly, such realistic career expectations are shown to be persistent over time and greatly influence young people's later educational and occupational attainment [17–19]. However, the literature concerning health career expectations has primarily concentrated on the specialty choice of university medical students. It mainly used regional or national datasets, making its findings difficult to generalize to larger populations and different countries [20, 21].

Social cognitive career theory (SCCT) is a predominant conceptual framework explaining individual educational and occupational interests, choices, and performance using cognitive-personal variables (self-efficacy, outcome expectations, and goals) and contextual experiential variables (gender, SES, academic ability, and other environmental factors) [22]. It highlights the importance of integrating personal inputs and contextual factors to explain individual career choices. Many empirical pieces of evidence support the effectiveness of SCCT conceptual models across different cultures [23, 24]. Nevertheless, there is a call for more empirical research on social, cultural, and economic environmental factors that significantly influence cognitive-personal variables and individual career choice behaviour [25]. The present study investigated how environmental factors might affect adolescents' health-related career expectations across different economies.

Concerning contextual influences, SCCT delineates two dimensions of environmental variables: objective and perceived aspects of the environment and distal and proximal environmental factors [25]. Although individuals are not passive repositories of environmental influences, they are potently affected by objective factors with or without awareness. Hence, we focused on the effects of the objective aspect of environmental factors, such as health expenditure and working conditions. Distal contextual factors, such as role models and family support, influence personal learning experiences. In contrast, proximal contextual factors, such as informal career contacts and discriminatory hiring practices, are influential when making educational and career decisions [25]. Since 15-year-olds do not need to make career choices immediately after graduation, this study concentrated on distal contextual influences on adolescents' health career expectations. In sum, we examined how national economic indicators, health working conditions, and personal background factors shaped adolescents' expectations for medical (doctor) and nursing careers.

Given the centrality of the health workforce in sustaining health systems and the considerable time and resources that will be devoted to training highly skilled health workers, the early planning and recognition of the health workforce potential in various economies are crucial. This study aimed to answer (a) how popular medical and nursing careers are among young people and (b) what is the relative importance of personal inputs and contextual factors in affecting adolescents' health career expectations. Only when these questions are answered can human resource planning departments and educational systems tailor their career guidance strategies and health training programs to strengthen students' health career interests and form determined health career expectations at an earlier stage.

## Methods

### Data

This study used the large-scale triennial Programme for International Student Assessment (PISA) dataset orchestrated by the Organisation for Economic Cooperation and Development (OECD). PISA uses (a) a two-stage sampling process with schools at the first stage and students at the second and (b) appropriate weights in analyses to ensure the national representation of 15-year-olds. Students' career expectations are available from 2000, 2003, 2006, 2015, and 2018. We included 61 economies with at least two recent cycles of career data, namely, PISA 2015 and 2018 (see details in Additional file 1: Table S1). The other corresponding economy-level indicators were obtained from the World Health Organization, the International Labour Organization, and the World Value Survey.

**Table 1:** Constructs and indicators used in the research as linked to social cognitive career theory (SCCT)

Constructs	Indicators	Descriptions	Sources	Analyses
Distal contextual factors				
National economy	GDP	GDP per capita	International Monetary Fund	
	Health expenditure	Health expenditure per capita	World Health Organization	Residualized to reflect the amount on top of the amount predicted by GPD
Working conditions	Salary	Average daily salary for health and social workers (in standardized US dollar)	International Labour Organization	Residualized to discount GDP effects
	Working hour	Average daily working hours for health and social workers	International Labour Organization	
	Safety	Average medical injuries per 10 000 health and social workers	International Labour Organization	
	Professional density	Number of doctors/nurses per 10 000 population	World Health Organization	
Proximal contextual factors				
Societal evaluation	Value income	Percentage of people who value income most when choosing a career	World Value Survey	
Cognitive-personal variables				
Gender	Girl preference	High scores indicate girls’ (vs. boys’) greater preference for health career		Multilevel Logistic Regression
SES	High SES preference	High scores indicate high-SES (vs. low-SES) students’ preference for health career		Multilevel Logistic Regression
Ability	High ability preference	High scores indicate high-academic ability (vs. low-academic ability) students’ preference for health career		Multilevel Logistic Regression

*GDP* gross domestic product, *SES* socioeconomic status

Many studies have used PISA to understand adolescents’ career interests and expectations, but they have mainly concentrated on science and teaching careers. For instance, using PISA 2006, Sikora and Pokropek showed that gender disparities in adolescents' science career expectations are larger in advanced industrial countries [26]. Guo used PISA 2015 to demonstrate that many Western countries succeed in attracting adolescents with high instrumental motivation to science-related careers regardless of their science achievement [27]. Furthermore, Han et al. investigated the important role of salaries, working hours, and societal respect in influencing adolescents’ teaching career expectations with PISA 2006. They found that the association differs for students with different capabilities. This study, to our knowledge, is the first to use PISA to explore adolescents’ interest in medical and nursing careers [28].

### Measures

Specifically, according to SCCT, we investigated (a) objective distal environmental factors: national economic development (gross domestic product [GDP]), health expenditures, and health working conditions (salaries, safety, working hours, and professional density); (b) perceived distal environmental factors (societal evaluation of a career); and (c) personal inputs (gender, SES, academic ability) in shaping adolescents' expectations for medical and nursing careers.

#### Adolescents' career expectations

This outcome variable was measured by a single open-ended question: "What kind of job do you expect to have when you are approximately 30 years old". The answers were coded using the International Standard Classification of Occupations 2008 (ISCO-08). We specifically focused on students' medical and nursing (and midwife) career expectations and compared them against all other career choices. All examined occupations required Skill Level 4, which entails a first degree issued at a higher educational institution for a minimum of 3–6 years of study.

#### Academic ability

The PISA assesses students' ability to apply their knowledge and skills in reading, math, and science to deal with real-life problems. Students' science and mathematics scores (*M* = 500, *SD* = 100), which are generally strongly related to students' medical/nursing career expectations, were averaged to become an indicator of students' academic ability (abbreviated as Sci below).

#### Socioeconomic status

Students' SES background was measured by an index integrating parents' highest level of education, parents' highest occupational status, and home possessions. The indices were designed to be comparable across economies [29].

#### Environmental factors

As one of our main interests was how various economy-level factors affect medical/nursing expectations, we obtained a set of economic and health-related environmental factors from reliable resources. The links between variables identified by SCCT and those used in this study are provided in Table 1. The economic development and health expenditure (abbreviated as "Economic" below) indicators were (a) gross domestic product per capita (GDP) [30] and (b) expenditure on health per capita (ExpendHeatlh; to provide a measure of money spent on health on and above predicted by the GDP; statistically, we used the residualized method so that it became the additional money spent on health beyond the amount predicted by the economy's GDP) [31, 32].

The health profession working conditions (HealthCon) were measured by (a) salary (average daily salary for health and social workers in standardized US dollars; statistically residualized by the GDP so that the GDP adjusted salary to be more comparable across low-/high-GDP economies) [32, 33]; (b) working hours (WorkHr, average daily working hours for health and social workers) [33]; (c) injuries (average injuries per 10 000 health and social workers) [33]; (d) density (number of doctors/nurses per 10 000 population) [31]; and (e) value income (VIncome; percentage of people who value good income most when looking for a job) [34].

#### Personal inputs

The differences in how students' background (SBkGd) might affect students' career expectations were measured by (a) girl preference (GirlPref; high when more girls expected to have medical or nursing careers); (b) high SES preference (HSESPref; high when more high SES students expected to have medical or nursing careers); and (c) high academic preference (HAcaPref; high when more high academic ability students expected to have medical or nursing careers).

### Data analyses

In estimating the impact of personal backgrounds (gender, SES, ability) in each economy, multilevel logistic regression models were adopted. Following previous research, the school average SES and the school average academic achievement (averaging reading, math, and science scores for each school) were controlled (statistically, the benefits of studying in a high SES or high academic school were removed) [35]. Specifically, Level 1 (Student) and Level 2 (School) are modelled as follows:

$${\eta }_{ij}=log\left[\frac{{\pi }_{ij}}{\left(1-{\pi }_{ij}\right)}\right] ={\alpha }_{0j}+{\beta }_{1j}\left(Female\right)+{\beta }_{2j}\left(SES\right)+{\beta }_{3j}\left(Sci\right),$$

$${\alpha }_{0j}={\beta }_{00}+{\beta }_{01}\left(Sch.SES\right)+{\beta }_{02}\left(Sch. scores\right)+{\epsilon }_{j}.$$

In the first step of PISA data analyses, for each economy, we examined the popularity of doctors and nurses and how individual students' factors (gender, SES, Sci ability) affected their expectations. In the second step using hierarchical regression, these students' characteristics (SBkGd, obtained from the first step) of each economy were used with their economic and HealthCon factors (obtained from publicly available information; see correlations in Table 2) to predict the popularity of medical and nursing expectations (from the first step).

**Table 2:** Correlations of medical and nursing expectations with various economic and health environments and student background factors

	Medical expectation	Nursing expectation
GDP	− .270^a^	.190
Health expenditure	− .162	.408^c^
Daily salary	− .080	.094
Daily work hours	.370^b^	− .280^a^
Injuries	− .216	− .004
Professional density	− .327^b^	.366^b^
Value income	.255^a^	− .135
Girl preference	.275^a^	− .060
High SES preference	.112	− .114
High academic preference	.062	.053

*GDP* gross domestic product, *SES* socioeconomic status

a = *p* < .05; b = *p* < .01; c = *p* < .001

In addition, we explored some nonlinear and moderating effects, specifically to examine the possibilities of (a) a bell curve (quadratic) relationship indicating that the average economies might have higher (or lower) medical career expectations than the richest and poorest economies and (b) the various factors having very different effects in low- and high-GDP economies (e.g., whether daily wages were important in low- but not in high-GDP economies). Our hierarchical regression analyses (see details in the Results section) showed that (a) the GDP effect on medical career expectations was curvilinear and (b) the effects of injuries (for doctors) and salary (for nurses) were different in low- and high-GDP economies.

## Results

**Fig. 1:**
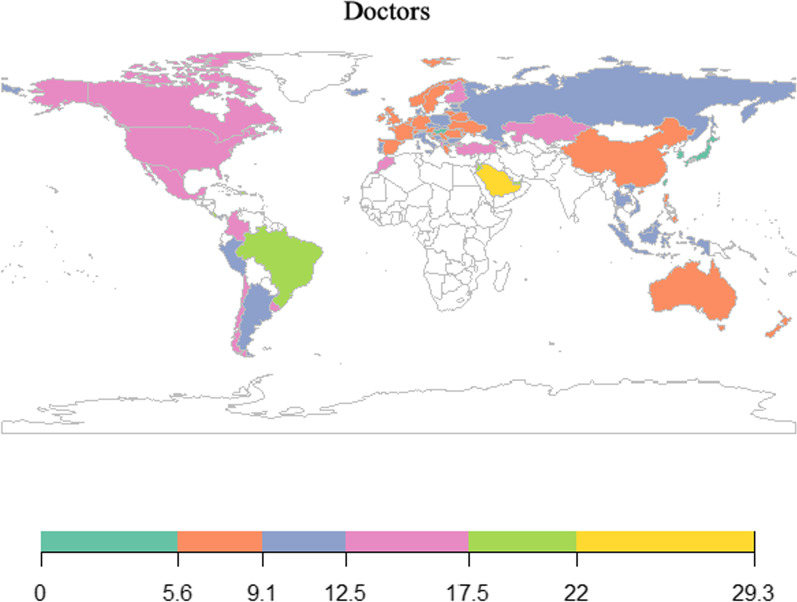
Percentages of adolescents expecting medical careers in 2018

**Fig. 2:**
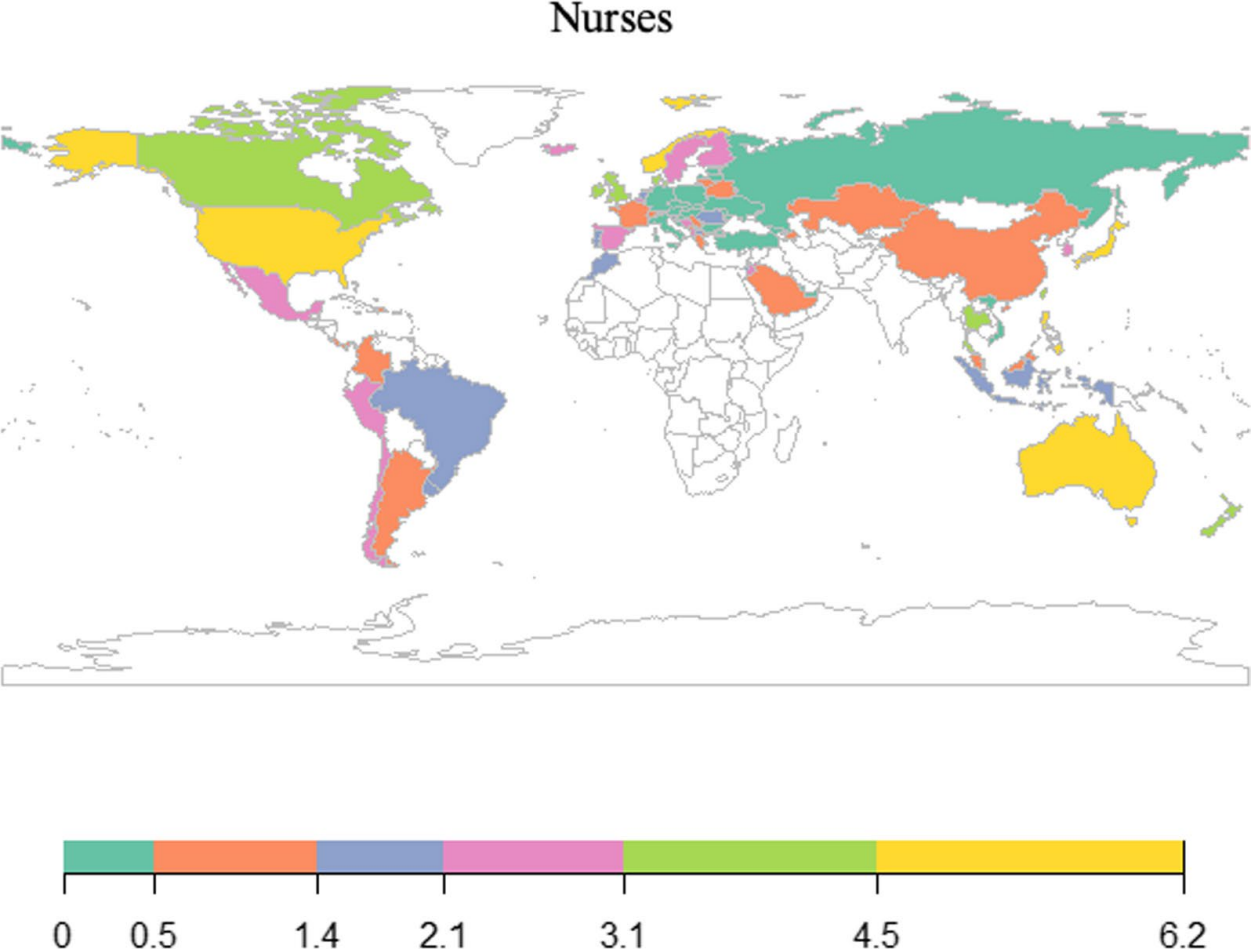
Percentages of adolescents expecting nursing careers in 2018

As shown, the relative percentages of adolescents expecting to pursue medical and nursing careers were quite different, sometimes even opposite, in the same economy (Figs. 1 and 2; Table 1). For medical careers, approximately 11% of young people in each economy expected to have such a career, and a large proportion were located in the Middle East and South America. For nursing, the overall percentages were lower than those found for medical careers. Only approximately 2% of adolescents expected to become nurses, and relatively more students were located in Australia, North America, and some Asian and European countries.

### Relative importance of economy, health working conditions, and student background factors

For medical careers, system-level factors were more critical, with economic factors accounting for 10% of the differences (mathematically, the variance) and the health environment adding another 21% of the students' career expectations. Student-level background added an additional 10% (Table 3, Models I, II, III). Similarly, for nursing, economic factors dominated and explained 21%, the health environment added 5%, and student background added only 1% (Table 4).

**Table 3:** Standardized beta values in hierarchical regression predicting medical career expectations with economic environment, health working conditions, and student backgrounds

	Economic I	Economic + HealthCon II	Economic + HealthCon + SBkGd III	Economic + HealthCon + SBkGd + GDP^2^ IV	Economic + HealthCon + SBkGd + GDP^2^ + inter’n V
GDP	− .278^a^	− .032	− .156	− .379	− .351
ExpendHealth	− .174	.039	.158	.205	.215
Salary		.081	.037	.073	− .017
WorkHr		.388^a^	.460^b^	.438^a^	.390^a^
Injuries		− .329^a^	− .518^c^	− .497^c^	− .574^c^
Doctor density		− .257^a^	− .250^a^	− .170	− .092
VIncome		.079	− .131	− .153	− .188
GirlPref			.203	.162	.191
HSESPref			− .002	.032	.024
HAcaPref			.274	.325^a^	.328^a^
GDP^2^				.173^a^	.137
GDP × Injuries					− .342^b^
Multiple R	.321	.561	.646	.679	.734
R^2^	.103	.315	.417	.461	.539
Adjusted R^2^	.072	.224	.300	.340	.423

*GDP* gross domestic product, *SES* socioeconomic status

a = *p* < .05; b = *p* < .01; c = *p* < .001

**Table 4:** Standardized beta values in hierarchical regression predicting nursing career expectations with economic environment, health working conditions, and student backgrounds

	Economic I	Economic + HealthCon II	Economic + HealthCon + SBkGd III	Economic + HealthConr + SBkGd + GDP^2^ IV	Economic + HealthCon + SBkGd + GDP^2^ + inter’n V
GDP	.208	.085	.107	.192	.117
ExpendHealth	.417^c^	.509^a^	.533^a^	.524^a^	.575^b^
Salary		− .271	− .256	− .259	.010
WorkHr		.023	− .023	− .010	.045
Injuries		.009	− .028	− .040	− .091
Nurse density		.158	.058	.030	− .120
VIncome		− .050	− .099	− .083	− .017
GirlPref			− .024	− .044	− .040
HSESPref			− .019	− .035	.000
HAcaPref			.109	.101	.142
GDP^2^				− .054	− .157
GDP × Salary					− .374^a^
Multiple R	.458	.506	.514	.518	.577
R^2^	.210	.256	.264	.269	.333
Adjusted R^2^	.182	.158	.117	.105	.167

*GDP* gross domestic product, *SES* socioeconomic status

a = *p* < .05; b = *p* < .01; c = *p* < .001

### Importance of specific factors

Based on SCCT, we focused on factors conducive to attracting more adolescents to medical and nursing careers. ***GDP.*** In the analyses involving the nonlinear effects, adolescents' medical career expectations in the low- and high-GDP economies tended to be higher than those in the middle-GDP economies (the quadratic term was positive but not consistently statistically significant in both Models IV and V).

#### Expenditure on health per capita

The extra government expenditure on health (on top of the GDP) was the most important and probably the only critical factor in attracting students to nursing careers among the various predicting factors examined in this study. However, extra health expenditure did not help doctors’ career expectations.

#### Working hours, injuries, professional density

Favourable health working conditions were found to be vital in attracting adolescents to medical careers but not to nursing. Economies with longer working hours, fewer injuries on the job, and fewer doctors in the population (lower doctor density, although not in Models IV and V) tended to attract more adolescents to medical careers.

#### Students' background factors

Whether an economy attracted more girls (or boys) or low (or high) SES students to medical and nursing careers did not affect adolescents' health career expectations in an economy. However, systems that could attract more high academic ability (HAcaPref) students to medical careers would in turn have more adolescents expecting medical professions, although this would not affect adolescents' choices of nursing careers.

### Combinational factors

Statistically significant combinational (moderation) effects were noted, and subsequent simple slope tests were conducted (Marsh et al. 2013). First, for medical careers, low injuries were critical to attracting more adolescents in high-GDP economies. In contrast, this factor was not influential in low-GDP economies (slope difference 0.684 for one standard deviation change of GDP). Second, for nursing, a high salary was comparatively more essential to attract students in low-GDP economies than in high-GDP economies (slope difference 0.748 for one standard deviation change of GDP).

## Discussion

To tackle global health workforce shortages, knowing the human resource potential is as vital as channelling current medical students to proper specialties and locations. Adolescence is a critical phase when educational and occupational interests, beliefs, and choices are formed and stabilized [36]. Understanding adolescents' health career expectations and the related influencing factors in various economies is indispensable to planning early and establishing a sustainable health workforce. The present study showed that North American countries do relatively well in attracting many adolescents who expect to have either a medical or nursing career. For many other economies, the proportion of students expecting to have a medical career was found to be quite different from those expecting to have a nursing career. However, the overall distribution of health career expectations did not resemble the professional density of doctors and nurses in each economy. This outcome likely reflects the limited number of medical degree places and stringent licensing requirements in many economies.

Guided by SCCT, we investigated the relative influences of personal inputs and contextual factors. We found that the local economy, health expenditure, and health working conditions account for the majority (one-third of the variance in choices) of adolescents' health career expectations. Specifically, conducive health working conditions (higher salaries and fewer injuries) attract more students to medical careers, while favourable economic conditions and high health expenditure contribute more to forming adolescents' nursing career expectations. In contrast, personal background, in terms of gender, social status, and academic ability, was found to explain much less of students' medical (10% of variance) and nursing (1%) career expectations. In this regard, government-level environmental support is more crucial than personal characteristics in determining adolescents' expectations for health careers.

### Medical career expectations

In particular, a safe work environment for health workers was found to be more critical for attracting adolescents to become doctors than remuneration. The generally high-ability students, potentially aiming to be doctors, were highly competitive and would opt for other highly rewarded and trendy careers when the health work environment was unattractive (e.g., high injuries). Thus, protecting health workers from medical injuries and other physical violence by patients' families is essential to attract adolescents to medical careers, especially in economically developed countries. Moreover, it was surprising to find that economies with longer working hours tended to attract more adolescents to medical careers, even when controlling for remuneration and work safety. The long working time probably reflects that the health profession is an extraordinarily challenging and specialized career that is highly valued and respected by the local community. The societal evaluation of health professions strongly influences young people's willingness to enter such careers [28].

The results suggest a curvilinear relationship between GDP and medical career expectations, indicating that medical professions are particularly attractive in both the highest- and the lowest-GDP economies. In economically developed countries, medical careers attract many high-ability students partly because they are well-paid, challenging jobs with high status [37]. Reports document that the average salary of doctors, including generalists and specialists, is substantially higher than the average wage for other occupations across OECD countries [37]. In developing economies, remuneration is vital in attracting adolescents to health careers because other careers might not be as lucrative. Prior studies have shown that professional conscience and supportive human resource management tools, rather than financial incentives, determine health workers' motivation in developing countries, especially in Africa [38]. As rising salaries could be less realistic in many underdeveloped countries, improving the societal evaluation of health careers as important, responsible, and respected could make doctors more popular in these regions.

### Nursing career expectations

Nurses and midwives are the backbone of maintaining global health systems and comprise more than half of the global health workforce [39]. Increasing adolescents' interest in nursing and midwifery careers is indispensable but not easy. Although girls from lower SES backgrounds generally expect to have nursing careers, we have shown that the impact of personal inputs is relatively trivial compared with contextual factors. In contrast to doctors, the influence of health working conditions (salaries, working hours, and safety) was not found to be deterministic in forming adolescents' nursing expectations. Notably, the government's additional expenditure on health (on top of the regular GDP allocation) was perhaps the most vital factor in enhancing adolescents' nursing career expectations, at least among the indicators explored. As labour market demands correlate with fiscal expenditure, a higher health expenditure can increase job vacancies, make nursing a safe career choice, and satisfy some adolescents' desire to care [40].

Nevertheless, a combinational (moderating) effect between nurses' salaries and GDP showed that higher income does not necessarily lead to adolescents' higher nursing expectations in economically developed countries. However, in less-developed economies, higher remuneration is critical in attracting adolescents to expect nursing careers. According to SCCT, financial incentives substantially affect adolescents' outcome expectations regarding nursing career choices and become a potent extrinsic motivation [22]. Higher salaries also provide strong environmental support for an individual to stay in nursing. Although previous studies have found that parental involvement in nursing and personal experiences with nurses underpins students' interest in nursing careers [41, 42], this study showed that higher health expenditure and higher remuneration for nurses, serving as distal environmental support, are also indispensable in promoting adolescents' nursing career expectations, particularly in developing countries. Countries with financial resources could invest in reducing medical school tuition fees, increasing full-time job opportunities, and improving their remuneration packages to fill the future shortages of nurses and midwives.

### Policy implications

Interventions have been designed to train, recruit, retain, distribute, and manage health workers to achieve adequate universal health coverage [7]. Based on evidence-based health workforce projection, increasing medical school capacity, and attracting adequate and qualified candidates are prerequisites. Outreach programs and funding are accessible ways to increase the pull factors of students, but strategies can differ for medical and nursing careers in different economies. In wealthy nations, many high-income professions are available for academically high-achieving students; thus, a safe work environment and the professionalism and challenge of medical careers are more important than remuneration in attracting adolescents. Comparatively, for nursing careers, higher health expenditure and better salaries could be more attractive to prospective candidates in developing economies.

The proportions of adolescents expecting health careers were not found to be parallel to the professional density in the specific economies. This outcome could be due to the international migration of health workers, limited medical training places, and other local licensure restrictions. For advanced economies that lack prospective medical students, understanding context-specific push and pull factors aiming to reduce outmigration is a potential solution. Increasing in-migration through formal bilateral partnerships with less-developed countries is another common strategy, which has been successfully practised in Canada, Germany, and Finland [43]. However, for lower-income sending countries already struggling with insufficiently trained medical staff, excessive outmigration can aggravate local human resource shortages, which codes of ethics should protect. To build a stronger domestic health workforce in developing economies, if extra financial support is unattainable, then improving the societal evaluation of the health profession is essential. Improving health workers' social status and respect would hopefully improve their popularity among young people.

## Limitations

This study used country-level indicators to investigate adolescents’ health career expectations which showed a general trend across examined 61 economies. However, this method neglects contextual-specific influences within each economy. To develop effective strategies for improving human resources for health planning, further analyses are needed for each economy using individual-level (student-level) data. In this way, unique associations between young people’s career expectations and different environmental and personal influencing factors will be found in each country.

## Conclusions

The persistent global shortage of doctors and nurses is hindering the achievement of universal healthcare coverage by 2030, and the largest deficit of health workers is found in low- and middle-income countries. It is thus essential to understand the health workforce potential in various economies and the associated influencing factors. The analyses of data from 61 economies have provided important results and policy implications. (a) Adolescents' expectations for medical and nursing careers vary substantially in different economies and do not resemble the professional density distribution. The international migration of health workers has been common, but we must proceed cautiously. (b) Health expenditure and health working conditions are the most deterministic factors. In contrast, personal background factors (gender, SES, and academic ability) play a less critical role in explaining adolescents' medical and nursing career expectations. Thus, more attention should be given to understanding system-level context-specific push and pull factors in attracting adolescents. (c) More students expect to become doctors in higher- and lower-GDP economies than in average GDP economies. Thus, middle-income countries must expand their outreach and financing programs to attract more students to health careers. (d) Work safety matters more in attracting adolescents to medical careers in wealthy nations, while remuneration is more crucial in attracting students to nursing careers in lower-income countries. For economies to widen the pool, contextual system-level influences on adolescents' health career expectations should be taken more seriously when making effective human resource strategies.

## Supplementary Information

**Additional file 1: Table S1.** Proportions of adolescents expecting medical and nursing careers in 61 economies in 2018.

## Data Availability

The datasets analysed during the current study are available in the Program for International Student Assessment (PISA) repository. [https://www.oecd.org/pisa/data/].

## Abbreviations

GDP: Gross domestic product
OECD: Organisation for Economic Cooperation and Development
PISA: Programme for International Student Assessment
SCCT: Social cognitive career theory
SES: Socioeconomic status
WHO: World Health Organization
