# Supplementary material for: Attracting adolescents to become doctors and nurses: differential importance of personal and environmental factors in 61 economies
Source: Hum Resour Health. 2023 May 15;21:40. doi: 10.1186/s12960-023-00823-7 (PMC10183684; doi:10.1186/s12960-023-00823-7)
Supplement: Supplementary file 1 — Additional file 1: Table S1. Proportions of adolescents expecting medical and nursing careers in 61 economies in 2018. [file 12960_2023_823_MOESM1_ESM.docx]

**Additional file 1 :Table S1** *Proportions of adolescents expecting medical and nursing careers in 61 economies in 2018*

| **Economies** | **Doctor** | **Nurse** |
| --- | --- | --- |
| Australia | 8.2 | 4.9 |
| Austria | 7.1 | 0.3 |
| Belgium | 7.8 | 2.7 |
| Brazil | 18.8 | 2 |
| Bulgaria | 11.4 | 0 |
| Canada | 14.9 | 4.5 |
| Chile | 15.9 | 3.1 |
| Chinese Taipei | 5.2 | 3.9 |
| Colombia | 15.9 | 1.1 |
| Costa Rica | 21 | 1.1 |
| Croatia | 7.2 | 0.4 |
| Czech Republic | 9.5 | 0.3 |
| Denmark | 10.1 | 3.6 |
| Dominican Republic | 18.2 | 1 |
| Estonia | 9.6 | 0.3 |
| Finland | 13 | 2.4 |
| France | 7.3 | 1.1 |
| Georgia | 14.1 | 0.4 |
| Germany | 7.1 | 0 |
| Greece | 9.1 | 0.9 |
| Hong Kong | 5.6 | 4.5 |
| Hungary | 5 | 0.5 |
| Iceland | 10.4 | 2.2 |
| Indonesia | 11.3 | 1.9 |
| Ireland | 6.2 | 4.1 |
| Israel | 15.3 | 1.9 |
| Italy | 9.9 | 0 |
| Japan | 3 | 6.2 |
| Jordan | 22 | 2.8 |
| Korea | 3.8 | 2.3 |
| Lebanon | 25.7 | 1.4 |
| Latvia | 9.8 | 0.1 |
| Lithuania | 8.9 | 0.7 |
| Luxembourg | 7.1 | 0 |
| Macao | 9 | 1.2 |
| Malta | 7.9 | 1.9 |
| Mexico | 15.8 | 2.4 |
| Moldova | 8.8 | 0.3 |
| Netherlands | 6.4 | 1.7 |
| New Zealand | 8.6 | 3.5 |
| Norway | 7.5 | 5.7 |
| Peru | 10.1 | 2.8 |
| Poland | 9.4 | 0.3 |
| Portugal | 9.3 | 1.9 |
| Qatar | 24 | 0.3 |
| Romania | 9 | 1.8 |
| Russian Federation | 10.4 | 0.4 |
| Singapore | 12.5 | 2.8 |
| Slovak Republic | 10.2 | 0.2 |
| Vietnam | 11.8 | 0.5 |
| Slovenia | 4.7 | 1.6 |
| Spain | 8 | 2.4 |
| Sweden | 7.4 | 2.3 |
| Switzerland | 6.8 | 0.8 |
| Thailand | 12.1 | 4.3 |
| United Arab Emirates | 21 | 0.3 |
| Turkey | 17.5 | 0.3 |
| United Kingdom | 6.7 | 3.8 |
| United States | 15.9 | 5.7 |
| Uruguay | 14.2 | 1.5 |
| B-S-J-Z (China) | 8.8 | 1.3 |
